# Supplementary material for: The Network of Cancer Genes (NCG): a comprehensive catalogue of known and candidate cancer genes from cancer sequencing screens
Source: Genome Biol. 2019 Jan 3;20:1. doi: 10.1186/s13059-018-1612-0 (PMC6317252; doi:10.1186/s13059-018-1612-0)
Supplement: Supplementary file 1 — Figure S1. Schema of the NCG database. (PDF 338 kb) [file 13059_2018_1612_MOESM1_ESM.pdf]

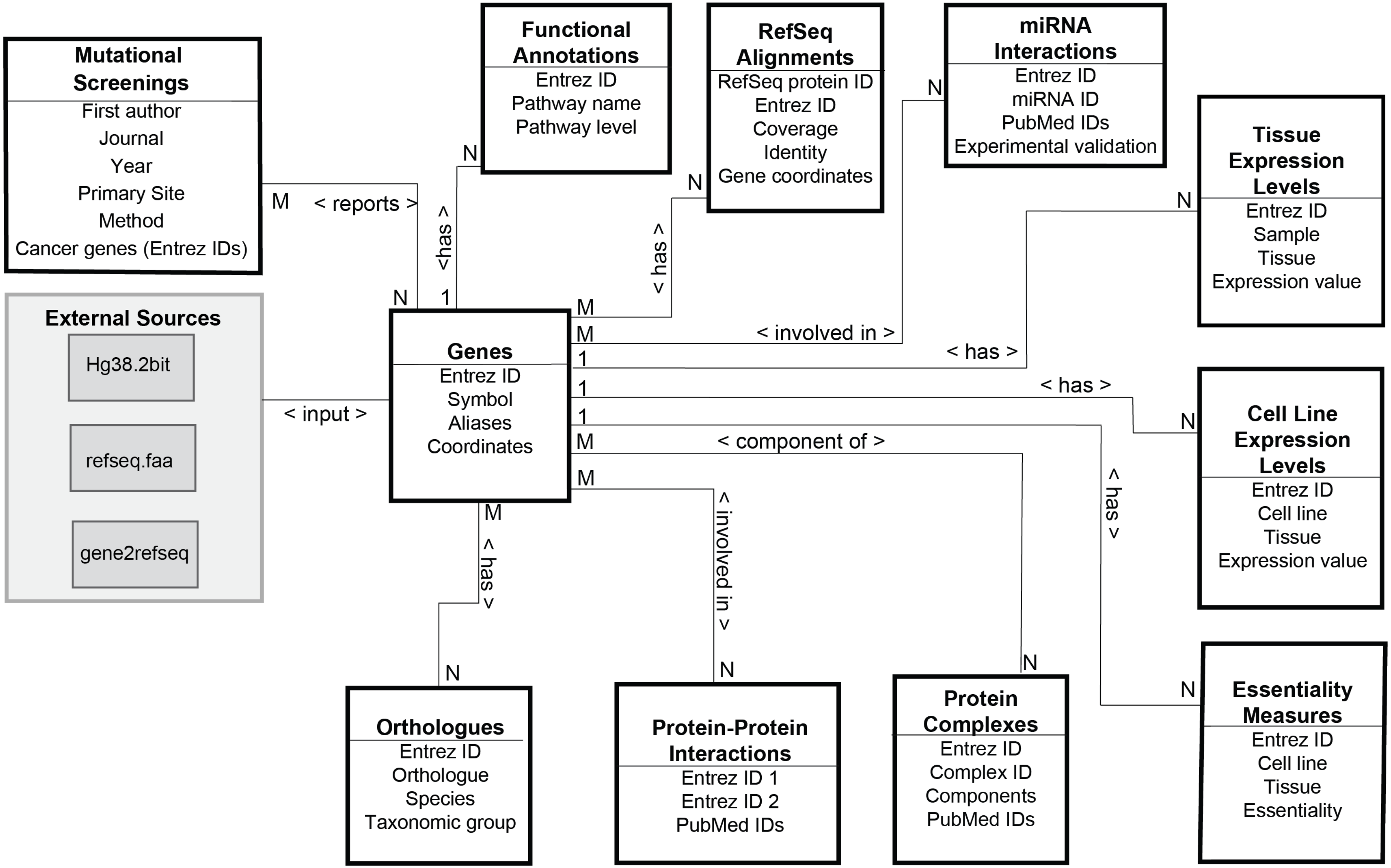

Mutational Screenings
First author
Journal
Year
Primary Site
Method
Cancer genes (Entrez IDs)
M
< reports >
N
Functional Annotations
Entrez ID
Pathway name
Pathway level
N
<has >
1
RefSeq Alignments
RefSeq protein ID
Entrez ID
Coverage
Identity
Gene coordinates
N
< has >
M
miRNA Interactions
Entrez ID
miRNA ID
PubMed IDs
Experimental validation
N
< involved in >
M
Tissue Expression Levels
Entrez ID
Sample
Tissue
Expression value
N
< has >
1
External Sources
Hg38.2bit
refseq.faa
gene2refseq
< input >
Genes
Entrez ID
Symbol
Aliases
Coordinates
< has >
1
N
Cell Line Expression Levels
Entrez ID
Cell line
Tissue
Expression value
1
< component of >
M
< has >
M
< involved in >
M
< has >
N
Orthologues
Entrez ID
Orthologue
Species
Taxonomic group
N
Protein-Protein Interactions
Entrez ID 1
Entrez ID 2
PubMed IDs
N
Protein Complexes
Entrez ID
Complex ID
Components
PubMed IDs
N
Essentiality Measures
Entrez ID
Cell line
Tissue
Essentiality

Additional File 1: Figure S1. Schema of the NCG database

Entity-relationship diagram indicating one-to-many and many-to-many relationships between genes and other entities in the NCG database. The external source files used to generate the Genes entity are shown in grey.
